# Computational Universalism, or, Attending to Relationalities at Scale

**DOI:** 10.1177/03063127251345089

**Published:** 2025-07-14

**Authors:** Francis Lee, David Ribes

**Affiliations:** 1 Media Technology, Södertörn University, Stockholm, Sweden; 2 Science, Technology & Society, Chalmers University of Technology, Gothenburg, Sweden; 3 Human Centered Design & Engineering, University of Washington, Seattle, WA, USA

**Keywords:** universalism, computation, data, algorithm, platform, domain

## Abstract

The social sciences and humanities have increasingly adopted computational terminology as the organizing categories for inquiry. We argue that by organizing research around vernacular computational objects (e.g. data, algorithms, or AI) and divided worldly domains (e.g. finance, health, and governance), scholars risk obscuring the universalizing practices and ambitions of computation. These practices seek to establish new relationalities at unprecedented scales, connecting disparate domains, circulating resources across boundaries, and positioning computational interventions as universally applicable. Drawing on intellectual traditions that inspect the fixity of universalizing claims, we problematize the easy adoption of computational categories and argue that they serve as epistemic traps that naturalize the expanding reach of computational universalism. Instead of accepting the hardened categories of our interlocutors, we propose attending to the partial, effortful, and often contested work of translation and commensuration that enables computational actors to position themselves as obligatory passage points across all domains. This approach reveals not only the remarkable achievements of computational relationalities at scale but also their exclusions, betrayals, and partialities. Our intervention aims to spur perspectives that examine how computational actors parse both technical objects and social worlds to advance universalizing ambitions while simultaneously obscuring the enormous labor required to maintain these divisions and connections.

A characterizing feature of contemporary computation is the production of novel relationalities at ever increasing scales: technologies that work here and there, resources that circulate anywhere and preserved for anywhen, connecting this lifeworld to that, all undergirded by common and compatible architectures. Meanwhile social scientific and humanistic inquiry of late seems to have devoted itself to computation in its constituent parts: tidy computational objects and divided worldly domains—just as our interlocutors portray them. It appears that even while computational actors work to capture all phenomena, circulate their objects anywhere, and position themselves to intervene in any domain, social science and humanistic inquiry has fractionated into computation’s component parts, organizing investigation around the elements of which computational actors say the world is made.

For many decades the pressures to adopt computational discourse have been overwhelming, but it is only recently that social scholarship has been in danger of reorganizing itself according to the words and ideas of computation. What is so curious is that just shortly before, Science and Technology Studies (STS) firmly rejected the fixity and totality of universalizing terms, instead seeking to bring other concerns to bear, whether political, epistemological, or both. In its formative moments STS sought to position itself laterally to the worlds that scientists and technologists produced, such as in the construction of facts (Haraway, 2003), the cultures of knowledge production (Knorr Cetina, 1999), the boundaries of science (Gieryn, 1983), or the classification of worlds (Bowker & Star, 1999), in all cases treating facts, knowledge, boundaries, and classifications as achieved and negotiated outcomes, thereafter sustained and redrawn with interest.

Our argument is that by approaching computation via categories defined by its actors—computational objects such as data, algorithm, and platform, or worldly domains such as health, governance or finance—we run the risk of missing the universalizing ambitions and practices that aim to interconnect and intervene, independent of all domains, everywhere and everywhen, internationally, globally, universally. This paper is a problematization and announcement of studies to develop an empirical and theoretical reparative to this regretful state of affairs.

We come to this argument from two trajectories.

On the one hand, we have observed how algorithms, data, software packages, and virtually any other computational object is engineered so as to travel: algorithms developed in the ecological sciences but that are then used to predict the taste of music listeners, equations representing earthquakes that are then used to predict crime, or data generated in biology but now circulating in physics too (Benbouzid, 2017; Brabazon & O’Neill, 2006; Leonelli & Tempini, 2020; Seaver, 2022). We have observed how engineers strategically work to develop techniques, tools, organizational forms, and technical systems that aim to be relevant in all places, now and later, casting computation as the universal means that will converge all representation and perform all analyses.

On the other hand, we have observed how large swaths of social scientific studies are following a seemingly converse trajectory. Programmatic papers in sociology, STS, and anthropology have called for the study of algorithms (Barocas et al., 2013; Ziewitz, 2011), platforms (Gillespie, 2010), data centers (Burrell, 2020), and datasets (Thylstrup et al., 2022)—each approached on their own even while always certainly pointing askance, analytically offstage, to the others. What unifies all these discrete research programs is that they are all organized around the technical objects of computation.

This is not to say that the move to attend to the objects of our informants has not been productive. We, *nostra culpa*, have done plenty of it too (Lee & Björklund Larsen, 2019; Ribes, 2017). The studies of algorithms, AI, data and platforms have produced many valuable insights about computational assemblages, rich insights for recognizing the practices and materialities of what are often reduced to technology and representation (boyd & Crawford, 2012; Dourish, 2016; Gitelman, 2013; Seaver, 2017; Thylstrup et al., 2022), attending to knowledge, science, and public contestation (Borgman, 2017; Edwards, 1999; Leonelli & Tempini, 2020), and to the power, ethics, biases, and governance of information (Barocas et al., 2013; Benjamin, 2019; Burrell & Fourcade, 2021; Hoeyer, 2023; Jaton, 2021; Kaun & Masso, 2025).

But by organizing around the names that the actors have given their objects, we are creeping towards naturalizing—rendering mundane, boring, unremarkable—how far the universalizing capacities of computing have come.

**Table:** 

**How to speak about ‘computing’?** We do not seek to wholly disavow the languages of computing, nor to develop a metalanguage that will break all links with the past. Data, algorithm, and domain are not banned by us. We simply wish for them not to serve as organizing categories for our fields. It is for this reason that we have strategically chosen to retain the modifier ‘*computational*’ *coupled with universalism.* Computing already serves as a recognizable umbrella term within both information technology discourse *and* in popular culture. It was just a short century ago that computation referred to a particular sort of gendered human labor, before ‘the computer boys took over’ (Ensmenger, 2012). But computation as a word isn’t quite so hot anymore, and perhaps it is even a little drab. The steaming vernaculars of the day all evoke breaks—‘*unsupervised*’ machine learning, ‘*large*’ language models—and new technical terms posit revolutions by the day. But words like computation and even data, algorithm, and domain, can still help us talk together about the things we care for. Just not as the central objects organizing investigation.

Here we seek to clear the way, first by sounding the hollows (pace Nietzsche) of received computational objects and divisions between worldly domains, and then attending instead to the making of heterogeneous and partial assemblages that stretch across time, place, societies and cultures, traveling on fibrous tendrils that seek to touch all.

The vast relationalities of computing are remarkable, unlikely, and transformative—but also troubling and worrying. The very basis of contemporary computation is to be transversal—‘interoperable’, ‘containerized’, ‘modular’– all these serving as the vernacular markers for the means by which computational actors seek to scale their practices to become universal.

As we will argue, seemingly discrete computational objects and tidy domain divisions are all about translation (Callon, 1986), commensuration (Espeland & Stevens, 1998), and partial relation (Strathern, 2004). We must attend to the enormous (also mundane, vernacular, and long-standing) work of generating computational relationalities at scale—acts of parsing and distinction that are conducted in tandem with travel and interconnection.

As Lee (2021, p. 67) notes in relation to the vernacular objects called algorithms, ‘we have fallen into an epistemic trap that delineates, stabilizes, and delimits our objects of study as well as our analytical problems’, while Suchman (2023) reminds us that the very invocation of ‘artificial intelligence’ risks a ‘hardening of the categories.’ Here we extend these arguments more widely, and seek to add friction to the epistemic trap of hardened categories, and call attention to specific, local instantiations *as well as* the ambitions and practices that seek to achieve universalizing relationalities at enormous (and growing) scales.

For fields such as STS, along with its travelling companions such as postcolonial studies, anthropology, or human geography (Mbembe, 2001; Thrift, 2008; Trouillot, 2003), there is nothing new about such universalizing claims. Unpacking universal ambitions has been the bread and butter of the sociology, philosophy, and history of science, such as by questioning ‘the unity of science’, the arrow of technological determinism, or the ‘bedrock of reality’ (Bijker, 1995; Galison, 1997; Smith & Marx, 1994). To such claims, allied fields have developed their own conceptual responses that have emphasized heterogeneity, locality, and circulation, via concepts such as the assemblage (DeLanda, 2006), the actor-network (Callon, 1986), natureculture (Haraway, 2003), or sociotechnical systems (Hughes, 1989), to name but a few. There is a great deal of nuance across those terms—varying traditions and goals—but they share a common rejection of the singularity and coherence posited by advocates of universal claims, instead calling attention to how things are drawn together via partial connection under conditions of continuous flux and contestation.

This article’s primary goal is to unseat the categories of computation as the starting points for the social study of the digital. In this, we cannot yet offer a full methodological reparative for inspecting the vast scales of computational universalism (really, we don’t yet have it; it is our project, and this paper serves as an invitation to participate) but in the discussion and conclusion we seek to breathe life into two matters of concern and associated methodological sensibilities that only recently have been sidelined: (i) inspecting partial relationalities at scale, and (ii) attending to translations, equivalencies, and betrayals, nodes and landscapes composed of concentration, circulation, absence, and exclusion.

## Computational universalism, then and now

Computational universalism is not new, it is not a distinct feature of ‘our digital society today’ even if now it is intensified and vast. Computational universalism starts well before our digitally networked era. After all, J.C.R. Licklider already envisioned an Intergalactic Network in the 1960s, leaving behind the tinyness of G.H. Wells’ World Brain or the modesty of de Chardin’s global noosphere (Licklider, 1963; Teilhard de Chardin, 1955; Wells, 2021). But the history of computational universalism stretches longer still, such as to statistics as the science that finds the stochastic in all, and to parallel efforts to gather total collections of peoples and oceans and trees (Hacking, 1990; Igo, 2007; Strasser, 2019).

Writing about the history of the early statistical experts in the 19th century, Gerd Gigerenzer and colleagues observe that:

[T]he mathematical statistician has become a universal expert, whose specialty is not so much a subject matter as a method of inference applicable to all subject matters. (Gigerenzer et al., 1989, p. 69)

Like data science today, statisticians of yesteryear recognized no limits for their tools, seeing them as equally applicable to every part of the world. They did not seek one distinct calculative device for suicide (Durkheim, 1951), another for agriculture or beer brewing (Fisher, 1935), and still another for war (Mindell, 2002). Instead, they all came in the form of the universal expert, who sought to bring an ‘independent’, ‘agnostic’ or ‘invariant,’ method to any and all subject matters at once (Ribes et al., 2019).

As with the universal expert of statistics, our call to inspect computational universalism does not especially distinguish the statistician from the mathematician, nor the computer or data scientist—indeed, they often arrive as a team, they are of a kind, or at least kin. Just as with computational techniques today, the quantifying (Porter, 1995), propositional (Carnap, 1969) or axiomatizing (Steingart, 2023) techniques of mathematics, logical empiricism, or statistics recognized no object, world, or domain distinctions: They sought formalisms which apply to all.

As Bowker (1993) notes in his inspection of cyberneticist’s universal strategies, systems engineers, too, sought to develop a common language that would be applicable in all cases and to all topics. In tandem, they also sought to articulate a new division of expert labor across academy and industry which, crucially, positioned themselves to act as the obligatory passage point for an interdisciplinary and cross-sectoral congress:

Their claim for universality was supported by a new reading of human history […] this new reading was bolstered by the development of a new universal language [which] in turn [was] used to suggest the validity of a new division of labour within the sciences (Bowker, 1993, p. 108).

What could serve to unify across military aircraft, psychiatry and the social structures of  Papua New Guinea (Heims, 1991)? The answer was always information, systems, and feedback (Hayles, 1999), even if also always, when cyberneticists returned to aircraft, psyches and cultures, they built their models and machines in ‘domain specific’ ways.

What we are identifying in this paper is a direct continuation of what Bowker and Gigerenzer et al. have called out, only now intensified in discourse and materialized in global technical architectures. Both statistics and cybernetics sought to respecify all objects in terms of their own techniques and vocabularies, and to do so they cast all ‘domains’ as disparate and divided so as to better position themselves as the bringers of new unities, the key intermediaries for a technologically achieved common congress.

Today’s disciplinary descendants of statistics, axiomatics and cybernetics—such as data science, machine learning, cloud computing … whatever—have inherited and furthered these ambitions and practices at unprecedented scales. The universality and transversality of computing is a constantly emerging phenomenon: What it means to connect and represent is in motion; it displays multiple genealogies and relies on evolving technique. Mathematization, quantification, standardization, and classification all remain the practices of contemporary universalization (Berg & Timmermans, 2000; Monteiro et al., 2012), but so too are new (or less discussed) entrants such as commensuration, interoperation, and harmonization (Desrosières, 2000; Espeland & Stevens, 1998; Ribes, 2017). Universal ambitions are wily and demand watchful attendance; the social scholar cannot simply return once again to the technique and organizational forms which characterized universal ambitions of yore—there is new method and architecture that was not there before.

Universal ambitions have propelled many novel relationalities, now at unprecedented scales—spanning across societies, cultures, disciplines, and domains. These scales and transversalities are what we want to attend to.

## Finding computational universalism strange again

Data, algorithms and domains. Oh my.

-The Tin Man to Dorothy

Our motive for initiating this project is our sense that we—social scholars of the computational—need to better understand the functioning, consequences, and reality-effects of computational universalism; to broaden our view from data, algorithms, or platforms as ‘applied’ to a specific domain or in order to cross multiples of them, and instead to understand how these newfangled and sprawling collectives shape our world in tangled rhizomes or spindly networks of computing, enacted through relationalities at ever-increasing scales.

The analytical and methodological tendency to organize social scientific work by the vernaculars of computation go well beyond a prescription to be respectful of actors’ categories or to be sensitive to emic matters of concern—it is instead threatening to make us into analytical and methodological dopes, repeating and reifying *ad infinitum* the objects of our interlocutors (see Garfinkel, 1967; Lynch, 2012).

### Diagnosis 1: The epistemic trap of vernacular objects

Suchman (2023) has noted the danger of falling into the epistemic trap of making computational objects the lynchpin of social investigations, pointing to what she has called ‘the ‘uncontroversial thingness’ of ‘AI’’ (see also Lee, 2021; Muniesa, 2019). Suchman especially calls attention to ‘the stabilizing effects of critical discourse that fails to destabilize its objects’ (p. 3): i.e. in critical data, algorithm or platform studies, these terms and categories are momentarily situated and historicized, perhaps an etymology unearthed, or a view on their relations to capital and colony offered, but then, somehow, right back we go to data, algorithm, platform. We appear to be riding on a well-worn merry-go-round of criticizing the terms of computation, but these efforts are so dangerous because following these hearty criticisms these objects recur, reify, and then loom. These critiques offer false refuge, and we fear that instead they propel stability; an uncontroversial thingness!

For certain, there have been many past hints at what we are noting here. Gitelman (2013) has observed that ‘raw data’ is an oxymoron; Latour (1999, p. 42) has admonished us ‘never speak of ‘data’—what is given—but rather of ‘sublata’’; and Drucker (2011) has made a parallel prescription that ‘Capta is ‘taken’ actively while data is assumed to be a ‘given’ able to be recorded and observed’. Versions of these appeals to Gitelman, Latour, and Drucker appear in scores of articles on studies of ‘data’, ‘algorithms’ or ‘platforms’, but then those articles return immediately—often in the very next line of writing—to talk of data, algorithm, and platform, lending not even the smallest measure of momentum to these alternate parsings.

We seek to unseat these orderings of computation so as to ask new questions, questions at a different scale. Though in truth we are not yet sure. Perhaps the new questions will be a return to classic concerns of STS: such as frames, genealogies, classification, and boundaries. But almost certainly there should be new questions too, questions that match these unprecedented scales. But importantly, these questions and emerging concepts must be born out of *social scientific and humanistic* concerns and problematizations so that we can better understand the wide if uneven encroachment of computation on so many lives, things, phenomena …

Our point is that data, algorithm, domain—but also, digital twins, ML, LLMs ... all of these will sound dusty by the time we publish this paper; but soon there will be more, each glowing red hot—each risks becoming an epistemic trap and ontological lure in our quest to understand how actors disassemble and reassemble these vast and partial relationalities.

### Diagnosis 2: The epistemic trap of bounded worlds

‘Domain’, just as with data or algorithm, is a vernacular word. It precedes formal computing as a term to describe societal life as a series of distinct disciplines, fields, or sectors. But in computational circles the term ‘domain’ has a technical meaning: it names an object of inquiry and a target for intervention (Ribes et al., 2019). Computational actors do not only parse their technical objects but also our social worlds, slicing and dicing them into ‘domains’—say, one for health, another for business, and still another for governance.

In computational circles, demarcating a domain serves to craft a target for technological development. As Avnoon has vividly written, data scientists are *omnivorous* consumers in their hunt for any domain knowledge to capture, graph, or model (Avnoon, 2023); while Haigh and Ceruzzi (2021) have dubbed the computer a modern *alkahest*, the universal solvent. Crafting domains are how computational actors objectify the world so as to engage it, omnivorous; while domain distinctions are cast as obstacles to overcome, always with computation as the singular means to dissolve them.

Within computational circles there are a series of technical virtues that aim to reach beyond any domain, to all, sometimes dubbed ‘domain independence’ and sometimes ‘platform agnosticism’, and for a long century now just ‘axiomatics’. Each of these impels the development of technologies unrelated to any particular aspect of the world, even while applicable to any of it: document files that open on any operating system; data from ecology, reused in economics; search engines indifferent to topic; semantic formalisms that that promise to capture any meaning; algorithms that are equally valid for earthquake and crime prediction.

If such capacities at scale seem obvious, natural, or unremarkable, it is only because all the work of extending these accomplishments has been obscured and forgotten. Isn’t it curious that anthropology and physics are ‘clearly’ two distinct domains, and yet computation still asserts that both ‘have data’? How can there be a data-centric biology (Leonelli, 2016) if those data long ago found a use in physics (Kay, 2000)? We find it odd when critical scholars all agree about what is shared in common, and downright suspicious when that commonality is recurrently computational.

These translations between domains at scales unimagined just a few decades ago demand that we develop new sensitizing strategies, new ways of talking about these scalar multipliers across domains, disciplines, societies, and cultures.

Our point is that the unity of computational objects and divisions asserted for domains are poor starting points for any social or humanistic inquiry. To articulate a sphere of action or expertise—‘a boundary’ (Gieryn, 1983), ‘a user community’ (Woolgar, 1990), ‘an expert discipline’ (Forsythe, 1993)—is also partly to constitute it, to render it an object of inquiry or intervention. Computational actors render into practical and tractable chunks that which they seek to ‘capture’, ’represent’, or ‘graph’ whether in scruffy but detailed divisions, or neatly grouped chunks (Poirier, 2017).

It is at the intersection of these two diagnostics—computational objects treated as always already there, and divided domains that demand translation and unification via computation*—*that we feel the most analytic leverage has been lost for social and humanistic investigations. We are obscuring these actors’ most unrelenting efforts to universalize at scale. The division and unification of computational objects and worldly domains are the manifestation of a universal ambition, and a matter of ongoing and constant effort to achieve novel relationalities at scale. Connecting across objects and worlds is the *modus operandi* of computation.

## Attending to relationalities at scale

Our interest in computational universalism is an interest in relationalities at ever-increasing scales. It should be clear by now that in writing ‘scale’ we are not seeking to direct further attention to the social study of ‘big’ data or ‘large’ language models. Scale does not mean that which has already been declared as large in any simple fashion (Ribes, 2014). Instead, it means something akin to reach, more of it. We use the language of relationalities at scale to attend to translation and treason, unification and division, grouping and splitting, and to all that which authorizes travel, similarity, and difference, within and across objects and worlds.

To attend to these relationalities at mounting scales means recasting our analytical sensibilities. Rather than adopting computational terminology as the names for our objects of inquiry we are here insisting that social studies of computation must be reframed in other terms than the vernacular of our interlocutors—whatever terms!—so long as they lend analytic friction and leverage. The transversal goals of universal computing, practices which recognize no limits to their utility, are encoded into those very computational vernaculars. Those words, just as with ‘inter-networking’ or ‘inter-operability’, mark ambitions to function across all heterogeneities, even while the practices to establish ‘domain independence’ seek to position above or beyond any worldly sphere, ideally all of them at once.

Computer scientist Agre (1998), in calling for a ‘critical technical practice’, targeted his writing to the practitioners of AI, accusing them of too strong an assent to their own terminologies. Today it is instead the social and humanistic fields who must heed Agre’s warning. Computational words should serve as the conundrums of our inquiries: How should we approach computation as strange again? Just as the ‘Nacirema’ served as a distancing technique for attending to body rituals among ‘Americans’ (Miner, 1956), we need to attempt finding ways to distance ourselves from the vernacular of computing.

There is a social field called ‘data studies’, which names its object of inquiry ‘data’, and then its core process ‘datafication’! These computational terms serve humanistic and social studies to name the field of study, the object of inquiry, they name a final outcome, the practices and processes, *explanans* and *explanandum*! ‘Datafication’, ‘platformization’, and even ‘AI in the making’ all concede too much: as if many kinds of things really can just be reduced to one; as if a singular definitive logic already undergirds the dynamics of each; as if somehow these computational terms mark the certain joints of social and historical transformation.

Contrast these theoretical formulations with those that came only a few years before, such as framing and overflowing (Callon, 1998), commensuration (Espeland & Stevens, 1998), or generification (Pollock & Williams, 2009), terms also intended to engage mounting computation, but distinctly not named after it. Porter (1995) did not write of a ‘statisticalization’, instead he asked us to attend to trust in numbers. When Verran (2001) inspected competing Western and Yoruban logics, she never dubbed it all ‘numeration’ (as though number was already a single thing). And when those two forms of counting were brought together, it was not intermediated by a computer; instead, Verran asked us to attend to a bucket!

The travel, translation, stabilization, unification, and divisions of data, algorithm, platform, AI, in ‘one domain’ or ‘across domains’, should be recognized as goals, ambitions, sets of practices and evolving capacities, the objects of much work and materialization. Some of that work has already been invested and achieved years or decades ago, but much of it remains promissory, even metaphysical, and is observably populated by gaps and exclusions. What does not move, or remain stable, or carry forward? What is unavailable to the unity of inter-networking and inter-operation? What of the remaining inter-ference and inter-ruption (Serres, 2007)?

Strathern (2004) asks us to consider the partial relation, a truly disturbing concept for computation which prefers things to be linked or discrete, flowing or halted, edges to nodes or not. Following Strathern we seek to ask how actors work to establish partial relationalities at scale. Perennially, the answer is by crafting partial connections but then recasting those as universal totality. In attending to relationalities at scale we must remain aware of how one thing becomes the same as another—i.e. commensurable, comparable, compilable, collapsible—and how that which cannot is left aside as exhaust, overflows dismissed as externalities (Callon, 1998; Lee, 2024). The nice thing about attending to partial relations is that they can be there and not; we do not have to definitely decide, and instead we can attend to what is in, out, similar, different, or something else. Partial relations at scale is our committed method.

## Conclusion and a tentative agenda for research

How can we then render the work of dividing and connecting more concrete and particular, even while still attending to the vast relationalities that computation seeks to emplace? By siloing the world in ways handed to us by computational actors, by accepting hardened and stabilized categories, we run the risk of treating as epiphenomenal that which is the crux: generalized computational ontologies, proliferating (partial) connections, and mounting ambitions to connect ever more. That we would all approach data, algorithm, and domains as distinct and definitive objects is taking the path of least resistance, an ontological wish-fulfillment, acceding to the objects and fetishes of our interlocutors, always with further computation required to get further along.

In projecting our gaze into the future of social scholarship on computation we seek to inspect the making of unities and boundaries, attending to the scaled connections of our worlds. We have highlighted two parallel analytical problems for understanding the computational machines and practices of late: First, there is the risk of ontological closure, a misplaced concreteness as scholars reify the categories of computation, such as artificial intelligence, domain, data, algorithm, platform, digital twins …. By inadvertently naturalizing these entities and phenomena—or worse, presenting them as ‘thoroughly criticized’ but then reified just the same in our own lexicon—we are at risk of obscuring the work that propels these efforts to be transversal, accomplished quietly by defining discrete objects and divided worlds. In using these terms with such vigor we are at risk of propelling their universality. Second, the rote acceptance of the universal applicability of computational techniques to all worlds can render opaque the necessary intricate, often messy, always partial work of translating to make these universal entities hold across worlds, societies, cultures, disciplines, and domains.

As contrarian handholds, or sensitizing concepts (Blumer, 1954), to pursue this agenda we have begun to outline two matters of concern for inspecting computational relationalities at scale, two approaches that do not start from computation’s singular parts and our divided worlds that always call for unity via further computation.

First, render the practices of connecting, translating, and moving strange again. We must look at these practices of transversality and universality as unlikely and effortful outcomes and alliances, made possible by recurrent actors at particular times and places. The making of unitary computational objects and divided worlds should certainly be approached with curiosity, but they also display regularities, computation always cast as the means for overcoming difference, always the same rejoicing third in overcoming the constructed divisions of the world.

Second, we need to attend to the partialities and betrayals of these relationalities at scale. To achieve equivalency always already involves a loss and a betrayal even if there may also be a gain and growth (Law, 1997). The digital does not flow freely across the pulsating fiber-optic cables in superhighways of information because it wants to. It is an effortfully achieved outcome, and then fitfully achieved again. There are always frictions and breakdowns, actors left behind even as new ones achieve more than they could before. We need to attend to these penalties, breakdowns, exclusions and, yes, the truly remarkable novel capacities.

These are the objects of research we have sought to lay out for future social scientific and humanistic inquiry.

But even if our proposed matters of concern do not appeal, and we certainly admit they are partial, we hope that the reader will still take note of our first point: Social studies and humanistic inquiry must proceed without simply adopting (or even chasing) today and tomorrow’s computational vernacular. We need to break from the hardened categories of computation, which they offer as though on a platter as ready-made objects and domains for investigation. We must cultivate analytic objects that articulate matters of concern rooted in a theory and politics not yet defined.
